# Acquired salinity tolerance in rice: Plant growth dataset

**DOI:** 10.1016/j.dib.2020.106023

**Published:** 2020-07-15

**Authors:** Karthika Sriskantharajah, Shota Osumi, Sumana Chuamnakthong, Mami Nampei, Junrey C. Amas, Glenn B. Gregorio, Akihiro Ueda

**Affiliations:** a Graduate School of Biosphere Science, Hiroshima University, Higashi-Hiroshima, 739-8528, Japan; b Graduate School of Integrated Sciences for Life, Hiroshima University, Higashi-Hiroshima, 739-8528, Japan; c School of Biological Sciences, University of Western Australia, 35 Stirling Hwy, Crawley, WA 6009, Australia; d Institute of Crop Science, College of Agriculture and Food Science, University of the Philippines Los Baños, College, Laguna, Philippines; e International Rice Research Institute, DAPO Box 7777, Metro Manila, Philippines

**Keywords:** Dry weight, Plant growth, Rice, Salinity, Salt acclimation

## Abstract

This article describes the growth of 18 acclimatized and 11 non-acclimatized rice varieties grown in a hydroponic nutrient solution in a glasshouse. Four plants from each variety were grown under control conditions, salinity stress following control conditions (salinity), and salinity stress following acclimation (salinity/acclimation) conditions. Sampling was performed at the end of the salinity treatment (36 days of growth). Growth traits such as shoot and root biomass accumulation and lengths were measured for each variety, and the average was calculated using four replicates. This dataset may aid interested researchers in making comparisons with their data and further advance the research on the salinity acclimation process in rice.

Specifications table

**Table:** 

Subject	Plant Science, Salinity
Specific subject area	Varietal-based growth description on acquired salinity tolerant in rice
Type of data	Table
How data were acquired	Measurements of plants grown in a hydroponic nutrient solution (control), nutrient solution subjected to salinity (salinity) and salinity after acclimation (salinity/acclimation)
Data format	Raw
Parameters for data collection	Parameters used for salt acclimation ability of each variety were mean of the shoot and root biomass accumulation
Description of data collection	Seedings of twenty-nine rice varieties were grown in a hydroponic nutrient solution in a glasshouse. Three sets of four seedlings from each variety were maintained throughout the experiment (control, salinity and salinity after acclimation). Seedlings were harvested at 36 days of growth; lengths and biomasses were measured.
Data source location	Hiroshima University Kagamiyama, Higashi-Hiroshima, Hiroshima, Japan (34.397° N, 132.717° E)
Data accessibility	The data is provided in this article.
Related research article	Sriskantharajah et al., (2020) Contribution of two different Na^+^ transport systems to acquired salinity tolerance in rice. Plant Sci. https://doi.org/10.1016/j.plantsci.2020.110517

## Value of the data

• These data are useful because they provide baseline information on salt acclimation ability in rice and could be used by other researchers who need data on these varieties.
• Most of these varieties were obtained from NIAS World Rice Core Collection of the NARO-gene bank project, so the data enable other researchers to compare their data with this data and extend their analysis.
• These data represent an easy way to evaluate the salinity tolerant ability of susceptible rice varieties, thus could also be used in meta-analysis of salt-tolerant traits.
• The additional value of these data is easy collection; hence many varieties can be evaluated at the earliest stage of growth for salinity tolerance.

## 1 Data description

**Fig. 1:**
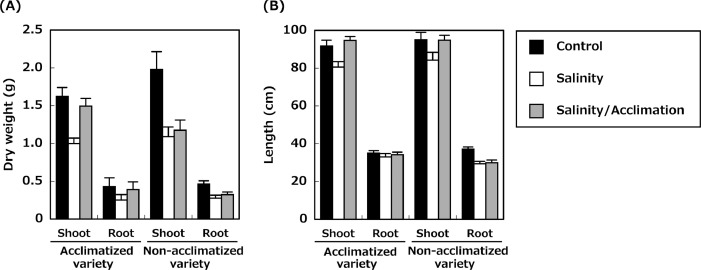
The average (A) shoot and root dry weights and (B) shoot and root lengths in 18 acclimatized varieties and 11 non-acclimated rice varieties grown under salinity stress conditions. The average was calculated for the shoot and root dry weight and shoot and root length under control, salinity stress following control conditions (salinity) and salinity stress following acclimation (salinity/acclimation). Data show the means of 72 and 44 plants ± S.E. of acclimatized and non-acclimatized varieties, respectively.

The dataset presented in this article (http://doi.org/10.1016/j.plantsci.2020.110517) provides details on the growth of 18 acclimatized and 11 non-acclimatized rice varieties grown hydroponically. The data from each variety were collected under three conditions: control, salinity, and salinity after acclimation. Growth measurements were taken at the end of 36 days of germination. The first dataset provides the shoot dry weight and length of acclimatized and non-acclimatized rice varieties, whereas the second dataset represents the root dry weight and length. In both cases, the first column is a variety name, and the next six columns are the measured traits of plants grown under the three different treatment conditions. Each column indicates the average of four samples along with the standard error of the measured trait. The same letter indicates no significant differences among the three treatments at *p* < 0.05. Table 1 presents the list of rice varieties with their taxonomic classification and country of origin. Fig.1 represents the (A) average shoot and root dry weight, and (B) average shoot and root lengths of both acclimatized and non-acclimatized varieties. Tables 2 and 3 represent the original data sets of dry weight and length of shoots and roots of both acclimatized and non-acclimatized varieties.

**Table 1:** List of acclimatized and non-acclimatized varieties available in the dataset.

No.	Variety	Country of origin	Subspecies	Type
**1. Acclimatized varieties**				
1	ARC 7291	India	Indica	Landrace
2	Bingala	Myanmar (Burma)	Indica	Landrace
3	BR28	Philippines	Indica	Breed
4	Chin Galay	Myanmar (Burma)	Indica	Landrace
5	Dianyu 1	China	Japonica	Breed
6	Hakphaynhay	Laos	Indica	Landrace
7	Hong Cheuh Zai	China	Indica	–
8	Kaluheenati	Sri Lanka	Indica	Landrace
9	Khao Nok	Laos	Tropical Japonica	Landrace
10	Khau Tan Chiem	Vietnam	Tropical Japonica	Landrace
11	Naba	India	Indica	Landrace
12	Neang Phtong	Cambodia	Indica	Landrace
13	Nepal 555	India	Indica	–
14	Padi Kuning	Indonesia	Indica	Landrace
15	Rambhog	Indonesia	Indica	Landrace
16	Shwe Nang Gyi	Myanmar (Burma)	Indica	Landrace
17	Tima	Bhutan	Tropical Japonica	Landrace
18	Vandaran	Sri Lanka	Indica	Landrace
**2. Non-acclimatized varieties**				
19	Anjana Dhan	Nepal	Indica	Landrace
20	Asu	Bhutan	Indica	Landrace
21	Deejiaohualuo	China	Indica	–
22	IR 58	Philippines	Indica	Breed
23	Jena035	Nepal	Indica	Landrace
24	Jinguoyin	China	Indica	Landrace
25	Kalo Dhan	Nepal	Indica	Landrace
26	Padi Perak	Indonesia	Tropical Japonica	Landrace
27	Radin Goi Sesat	Malaysia	Indica	Landrace
28	Tupa729	Bangladesh	Tropical Japonica	Landrace
29	Urasan 1	Japan	Tropical Japonica	Landrace

**Table 2:** Shoot dry weight and length of 18 acclimatized and 11 non-acclimatized rice varieties under control, salinity stress, and salinity stress after acclimation. Data represent the means of quadruplicates ± SE. The same letters indicate no significant differences at *p* < 0.05.

No.	Variety	Shoot dry weight (g)			Shoot length (cm)		
		Control	Salinity	Salinity/Acclimation	Control	Salinity	Salinity/Acclimation
**1. Acclimatized varieties**							
1	Bingala	2.04 ± 0.06^b^	1.35 ± 0.14^c^	2.52 ± 0.12^a^	114.5 ± 1.1^a^	95.8 ± 2.2^b^	92.5 ± 0.6^b^
2	Neang Phtong	0.74 ± 0.14^ab^	0.51 ± 0.01^b^	0.90 ± 0.04^a^	91.5 ± 1.3^a^	81.5 ± 1.3^b^	92.2 ± 5.0^a^
3	ARC 7291	1.19 ± 0.06^ab^	1.02 ± 0.06^b^	1.39 ± 0.08^a^	75.5 ± 3.0^b^	76.6 ± 2.7^b^	100.3 ± 3.0^a^
4	Dianyu 1	0.99 ± 0.01^b^	0.60 ± 0.02^c^	1.14 ± 0.01^a^	75.3 ± 7.7^b^	66.6 ± 1.8^c^	94.2 ± 2.9^a^
5	Khau Tan Chiem	1.84 ± 0.04^a^	1.27 ± 0.14^b^	1.92 ± 0.06^a^	100.8 ± 3.6^ab^	97.2 ± 1.8^b^	104.5 ± 1.5^a^
6	BR28	0.97 ± 0.03^a^	0.55 ± 0.04^b^	0.94 ± 0.06^a^	64.3 ± 9.2^b^	51.7 ± 9.3^c^	101.3 ± 10.8^a^
7	Vandaran	1.65 ± 0.05^a^	1.12 ± 0.02^b^	1.58 ± 0.1^a^	97.4 ± 6.4^b^	83.4 ± 3.8^c^	110.4 ± 2.3^a^
8	Hong Cheuh Zai	1.50 ± 0.09^a^	0.75 ± 0.09^b^	1.43 ± 0.15^a^	95.4 ± 4.8^b^	76.8 ± 2.8^c^	102.9 ± 5.3^a^
9	Tima	1.76 ± 0.13^a^	1.09 ± 0.07^b^	1.68 ± 0.18^a^	110.9 ± 2.2^a^	101.9 ± 1.6^b^	105.7 ± 0.3^a^
10	Rambhog	1.25 ± 0.13^a^	0.76 ± 0.04^b^	1.18 ± 0.08^a^	84.4 ± 3.1^a^	77.0 ± 2.7^b^	89.0 ± 2.9^a^
11	Hakphaynhay	1.61 ± 0.16^a^	1.08 ± 0.09^b^	1.45 ± 0.11^a^	98.1 ± 3.7^a^	87.5 ± 3.7^b^	91.1 ± 5.8^ab^
12	Khao Nok	2.14 ± 0.15^a^	1.34 ± 0.15^b^	1.91 ± 0.05^a^	101.4 ± 2.9^a^	81.7 ± 2.9^b^	94.5 ± 5.9^a^
13	Nepal 555	2.36 ± 0.17^a^	1.54 ± 0.02^b^	2.00 ± 0.28^a^	90.3 ± 1.5^a^	84.1 ± 10.3^ab^	79.5 ± 8.8^b^
14	Padi Kuning	1.02 ± 0.06^a^	0.58 ± 0.03^b^	0.83 ± 0.08^a^	101.1 ± 3.1^a^	85.2 ± 1.9^b^	85.5 ± 3.3^b^
15	Naba	2.12 ± 0.31^a^	1.07 ± 0.08^b^	1.61 ± 0.13^a^	77.4 ± 2.3^a^	61.7 ± 5.8^b^	81.3 ± 5.3^a^
16	Kaluheenati	2.00 ± 0.08^a^	1.33 ± 0.1^b^	1.69 ± 0.08^a^	88.8 ± 1.3^a^	74.0 ± 1.6^b^	89.5 ± 1.8^a^
17	Chin Galay	2.23 ± 0.04^a^	0.91 ± 0.04^b^	1.51 ± 0.07^a^	97.0 ± 4.2^a^	84.1 ± 4.9^b^	99.9 ± 3.8^a^
18	Shwe Nang Gyi	1.81 ± 0.02^ab^	1.07 ± 0.02^b^	1.20 ± 0.04a	87.9 ± 3.7^ab^	83.7 ± 0.7^b^	89.9 ± 1.8^a^
**2. Non-acclimatized varieties**							
19	Deejiaohualuo	1.36 ± 0.09^a^	0.75 ± 0.04^b^	1.00 ± 0.11^b^	94.8 ± 3.5^b^	81.7 ± 2.9^c^	101.4 ± 4.6^a^
20	Urasan 1	0.97 ± 0.03^a^	0.65 ± 0.03^b^	0.68 ± 0.04^b^	75.7 ± 1.4^b^	64.2 ± 2.6^c^	103.1 ± 1.5^a^
21	Anjana Dhan	1.85 ± 0.17^a^	1.17 ± 0.05^b^	1.27 ± 0.08^b^	88.0 ± 3.0^b^	84.6 ± 5.0^b^	104.9 ± 5.5^a^
22	Tupa729	3.10 ± 0.16^a^	1.52 ± 0.19^b^	2.02 ± 0.38^b^	106.7 ± 2.6^a^	94.4 ± 1.0^b^	105.9 ± 0.4^a^
23	Padi Perak	0.81 ± 0.08^a^	0.51 ± 0.02^b^	0.52 ± 0.06^b^	82.4 ± 5.5^b^	74.3 ± 7.0^b^	93.5 ± 5.1^a^
24	Radin Goi Sesat	1.71 ± 0.13^a^	0.84 ± 0.01^b^	1.08 ± 0.14^b^	95.9 ± 3.0^a^	91.9 ± 5.0^a^	84.2 ± 2.4^b^
25	Jena035	2.25 ± 0.24^a^	1.65 ± 0.04^b^	1.32 ± 0.15^b^	103.0 ± 0.6^a^	88.2 ± 0.7^a^	87.9 ± 3.9^b^
26	Jinguoyin	2.97 ± 0.41^a^	1.63 ± 0.22^b^	1.65 ± 0.11^b^	116.9 ± 1.4^a^	95.8 ± 1.8^b^	92.8 ± 1.3^b^
27	Kalo Dhan	1.92 ± 0.18^a^	0.83 ± 0.08^b^	1.04 ± 0.06^b^	102.0 ± 3.1^a^	89.6 ± 0.6^b^	99.1 ± 0.8^a^
28	Asu	2.93 ± 0.34^a^	1.55 ± 0.09^b^	1.55 ± 0.10^b^	102.8 ± 9.0^a^	103.1 ± 10.2^a^	86.7 ± 4.4^b^
29	IR 58	1.90 ± 0.51^a^	0.87 ± 0.06^b^	0.79 ± 0.10^b^	77.5 ± 4.4^a^	59.4 ± 1.8^b^	83.3 ± 1.9^a^

**Table 3:** Root dry weight and length of 18 acclimatized and 11 non-acclimatized rice varieties under control, salinity stress, and salinity stress after acclimation. Data represent the means of quadruplicates ± SE. The same letters indicate no significant differences at *p* < 0.05.

No.	Variety	Root dry weight (g)			Root length (cm)		
		Control	Salinity	Salinity/Acclimation	Control	Salinity	Salinity/Acclimation
**1. Acclimatized varieties**							
1	Bingala	0.54± 0.05^a^	0.35± 0.04^b^	0.66± 0.06^a^	43.2 ± 0.6^a^	37.7 ± 1.9^b^	42.2 ± 1.0^a^
2	Neang Phtong	0.20± 0.04^a^	0.12± 0.00^b^	0.22± 0.01^a^	36.9 ± 0.4^a^	32.0 ± 0.7^b^	31.8 ± 0.4^b^
3	ARC 7291	0.30± 0.01^b^	0.28± 0.03^b^	0.40± 0.04^a^	36.8 ± 0.2^a^	28.3 ± 0.1^c^	30.4 ± 0.5^b^
4	Dianyu 1	0.25± 0.01^b^	0.15± 0.01^c^	0.31± 0.03^a^	28.9 ± 1.0^a^	28.7 ± 1.2^a^	28.5 ± 0.8^a^
5	Khau Tan Chiem	0.44± 0.03^a^	0.23± 0.04^b^	0.42± 0.06^a^	33.3 ± 1.1^a^	32.0 ± 2.5^a^	35.1 ± 0.6^a^
6	BR28	0.22± 0.02^a^	0.14± 0.02^b^	0.21± 0.02^a^	29.8 ± 1.0^b^	26.0 ± 0.9^b^	34.4 ± 1.5^a^
7	Vandaran	0.37± 0.04^a^	0.26± 0.02^b^	0.40± 0.02^a^	30.4 ± 0.1^b^	28.9 ± 1.3^b^	34.7 ± 1.3^a^
8	Hong Cheuh Zai	0.36± 0.04^a^	0.17± 0.01^b^	0.32± 0.09^a^	34.6 ± 2.0^a^	25.8 ± 1.2^b^	30.9 ± 0.5^a^
9	Tima	0.46± 0.03^a^	0.23± 0.01^b^	0.39± 0.08^ab^	32.7 ± 0.8^a^	30.5 ± 0.2^ab^	27.7 ± 1.3^b^
10	Rambhog	0.25± 0.03^a^	0.17± 0.01^b^	0.25± 0.00^a^	34.7 ± 1.6^a^	34.0 ± 0.4^a^	35.4 ± 2.1^a^
11	Hakphaynhay	0.33± 0.00^a^	0.22± 0.01^b^	0.30± 0.03^a^	39.1 ± 1.5^a^	36.9 ± 1.2^a^	39.9 ± 3.4^a^
12	Khao Nok	0.80± 0.07^a^	0.42± 0.03^b^	0.72± 0.03^a^	38.7 ± 1.2^a^	47.9 ± 5.7^a^	37.3 ± 2.6^a^
13	Nepal 555	0.80± 0.03^a^	0.43± 0.02^c^	0.60± 0.07^b^	42.1 ± 0.7^a^	43.2 ± 3.2^a^	38.5 ± 2.0^a^
14	Padi Kuning	0.21± 0.01^a^	0.13± 0.00^b^	0.17± 0.02^ab^	35.0 ± 1.3a	37.4 ± 2.9^a^	37.0 ± 0.5^a^
15	Naba	0.58± 0.06^a^	0.30± 0.06^b^	0.47± 0.05^ab^	35.0 ± 0.6^a^	25.2 ± 0.4^c^	32.3 ± 0.6^b^
16	Kaluheenati	0.63± 0.01^a^	0.37± 0.03^c^	0.51± 0.05^b^	39.5 ± 0.5^a^	45.3 ± 3.4^a^	41.3 ± 1.3^a^
17	Chin Galay	0.48± 0.01^a^	0.20± 0.01^c^	0.28± 0.06^b^	22.2 ± 0.3^a^	16.0 ± 0.5^c^	19.0 ± 0.2^b^
18	Shwe Nang Gyi	0.48± 0.04^a^	0.33± 0.03^b^	0.40± 0.01^ab^	38.7 ± 0.6^a^	37.8 ± 2.0^a^	39.3 ± 0.5^a^
**2. Non-acclimatized varieties**							
19	Deejiaohualuo	0.34± 0.05^a^	0.17± 0.04^b^	0.23± 0.04^ab^	35.80± 0.7^a^	24.40± 1.9^b^	30.13± 0.3^ab^
20	Urasan 1	0.25± 0.02^a^	0.17± 0.01^b^	0.23± 0.02^ab^	31.60± 0.9^a^	31.37± 1.2^a^	32.80± 0.3^a^
21	Anjana Dhan	0.46± 0.01^a^	0.29± 0.02^c^	0.36± 0.02^b^	38.80± 0.8^a^	23.73± 0.4^c^	25.90± 0.2^b^
22	Tupa729	0.49± 0.04^a^	0.26± 0.07^a^	0.39± 0.08^a^	33.60± 1.0^a^	31.40± 2.7^a^	34.67± 5.9^a^
23	Padi Perak	0.31± 0.04^a^	0.15± 0.02^b^	0.17± 0.02^b^	33.40± 1.1^a^	29.10± 1.1^ab^	26.63± 2.2^b^
24	Radin Goi Sesat	0.43± 0.03^a^	0.19± 0.01^b^	0.24± 0.03^b^	34.70± 1.3^a^	24.57± 1.1^b^	24.47± 2.2^b^
25	Jena035	0.68± 0.09^a^	0.60± 0.02^a^	0.51± 0.06^a^	40.47± 0.2^a^	28.83± 0.9^b^	24.13± 0.5^c^
26	Jinguoyin	0.52± 0.08^a^	0.30± 0.02^b^	0.39± 0.06^ab^	36.37± 1.0^a^	29.47± 0.5^b^	28.33± 0.5^b^
27	Kalo Dhan	0.56± 0.12^a^	0.28± 0.03^b^	0.35± 0.03^ab^	45.40± 0.6^a^	34.20± 0.5^b^	32.50± 0.9^b^
28	Asu	0.68± 0.10^a^	0.40± 0.03^b^	0.46± 0.04^ab^	40.27± 1.5^a^	38.37± 2.1^a^	40.47± 0.1^a^
29	IR 58	0.37± 0.09^a^	0.21± 0.03^a^	0.21± 0.03^a^	38.10± 1.2^a^	27.57± 1.1^b^	29.20± 0.7^b^

## 2 Experimental design, materials and methods

The experiment was conducted in a glasshouse at Hiroshima University, Japan. The conditions of the glass house were 55% humidity, 19–27 °C day/15–20 °C night temperature and natural sunlight. Seeds of twenty-nine rice varieties selected from the World Rice Core Collection [1] (Table 1) were initially heat-sterilized at 60 °C for 10 min in a water bath, then surface-sterilized using 5% (v/v) sodium hypochlorite solution for 30 min, and finally rinsed thoroughly with distilled water. The seed germination process, seed transfer to Kimura-B nutrient solution, and the composition of the Kimura-B solution are provided in a related research article [2]. The nutrient solution was changed every 3 days, and the pH was maintained daily between 5.0–5.5.

Three sets of four seedlings from each variety were maintained throughout the experiment. One set received only the Kimura-B nutrient solution (control). In the second set, 1-week-old seedlings grown in the Kimura-B nutrient solution were acclimated with 1 mM NaCl (salinity/acclimation) for 2 weeks and then exposed to 50 mM NaCl for the next 2 weeks. In the third set, hydroponically grown plants were directly subjected to 50 mM NaCl (salinity) during the third week of growth and maintained for the next 2 weeks. The seedlings were harvested at the end of the salinity treatment (at 36 days of growth).

After harvest, the roots were thoroughly rinsed with distilled water and then gently dried with a paper towel [3]. The shoot and root lengths of each seedling were recorded using a measuring tape [4]. For dry weight determination, each seedling was divided into leaves, sheaths, and roots, which were then oven-dried at 70 °C for 3 days before being weighed [5]. Shoot dry weight was calculated by combining the dry weight of the leaves and sheaths.

The data were analyzed using a one-way ANOVA, and the differences between treatment means (*n* *=* 4) were compared the using Tukey-Kramer's multiple comparison test with SPSS, version 21 (IBM Inc., USA). Differences between the treatments were considered significant at *p* < 0.05.

## Ethics statement

Not applicable

## Declaration of Competing Interest

None.

## Appendix Supplementary materials

Image, application 1

## References

[1] Kojima Y., Ebana K., Fukuoka S., Nagamine T., Kawase M. (2005). Development of an RFLP-based rice diversity research set of germplasm. Breed. Sci..

[2] Sriskantharajah K., Osumi S., Chuamnakthong S., Nampei M., Amas J.C., Gregorio G.B., Ueda A. (2020). Contribution of two different Na^+^ transport systems to acquired salinity tolerance in rice. Plant Sci..

[3] Mekawy A.M.M., Abdelaziz M.N., Ueda A. (2018). Apigenin pretreatment enhances growth and salinity tolerance of rice seedlings. Plant Physiol. Biochem..

[4] Djanaguiraman M., Sheeba J.A., Shankaer A.K., Devi D.D., Bangarusamy U. (2006). Rice can acclimate to lethal level of salinity by pretreatment with subleathal level of salinity through osmotic adjustment. Plant Soil.

[5] Chuamnakthong S., Nampei M., Ueda A. (2019). Characterization of Na^+^ exclusion mechanism in rice under saline-alkaline stress. Plant Sci..

